# Hba1c, Blood Pressure, and Lipid Control in People with Diabetes: Japan Epidemiology Collaboration on Occupational Health Study

**DOI:** 10.1371/journal.pone.0159071

**Published:** 2016-07-20

**Authors:** Huanhuan Hu, Ai Hori, Chihiro Nishiura, Naoko Sasaki, Hiroko Okazaki, Tohru Nakagawa, Toru Honda, Shuichiro Yamamoto, Kentaro Tomita, Toshiaki Miyamoto, Satsue Nagahama, Akihiko Uehara, Makoto Yamamoto, Taizo Murakami, Chii Shimizu, Makiko Shimizu, Masafumi Eguchi, Takeshi Kochi, Teppei Imai, Akiko Okino, Keisuke Kuwahara, Ikuko Kashino, Shamima Akter, Kayo Kurotani, Akiko Nanri, Isamu Kabe, Tetsuya Mizoue, Naoki Kunugita, Seitaro Dohi, Japan Epidemiology Collaboration on Occupational Health Study Group

**Affiliations:** 1 Department of Epidemiology and Prevention, Center for Clinical Sciences, National Center for Global Health and Medicine, Tokyo, Japan; 2 Tokyo Gas Co., Ltd., Tokyo, Japan; 3 Mitsubishi Fuso Truck and Bus Corporation, Kanagawa, Japan; 4 Mitsui Chemicals, Inc., Tokyo, Japan; 5 Hitachi, Ltd., Ibaraki, Japan; 6 Mitsubishi Plastics, Inc., Tokyo, Japan; 7 Nippon Steel & Sumitomo Metal Corporation Kimitsu Works, Chiba, Japan; 8 All Japan Labour Welfare Foundation, Tokyo, Japan; 9 YAMAHA CORPORATION, Shizuoka, Japan; 10 Mizue Medical Clinic, Keihin Occupational Health Center, Kanagawa, Japan; 11 Furukawa Electric Co., Ltd., Tokyo, Japan; 12 Azbil Corporation, Tokyo, Japan; 13 Teikyo University Graduate School of Public Health, Tokyo, Japan; 14 National Institute of Public Health, Saitama, Japan; Baylor College of Medicine, UNITED STATES

## Abstract

**Aims:**

The control of blood glucose levels, blood pressure (BP), and low-density lipoprotein cholesterol (LDL-C) levels reduces the risk of diabetes complications; however, data are scarce on control status of these factors among workers with diabetes. The present study aimed to estimate the prevalence of participants with diabetes who meet glycated hemoglobin (HbA1c), BP, and LDL-C recommendations, and to investigate correlates of poor glycemic control in a large working population in Japan.

**Methods:**

The Japan Epidemiology Collaboration on Occupational Health (J-ECOH) Study is an ongoing cohort investigation, consisting mainly of employees in large manufacturing companies. We conducted a cross-sectional analysis of 3,070 employees with diabetes (2,854 men and 216 women) aged 20–69 years who attended periodic health examinations. BP was measured and recorded using different company protocols. Risk factor targets were defined using both American Diabetes Association (ADA) guidelines (HbA1c < 7.0%, BP < 140/90 mmHg, and LDL-C < 100 mg/dL) and Japan Diabetes Society (JDS) guidelines (HbA1c < 7.0%, BP < 130/80 mmHg, and LDL-C < 120 mg/dL). Logistic regression models were used to explore correlates of poor glycemic control (defined as HbA1c ≥ 8.0%).

**Results:**

The percentages of participants who met ADA (and JDS) targets were 44.9% (44.9%) for HbA1c, 76.6% (36.3%) for BP, 27.1% (56.2%) for LDL-C, and 11.2% (10.8%) for simultaneous control of all three risk factors. Younger age, obesity, smoking, and uncontrolled dyslipidemia were associated with poor glycemic control. The adjusted odds ratio of poor glycemic control was 0.58 (95% confidence interval, 0.46–0.73) for participants with treated but uncontrolled hypertension, and 0.47 (0.33–0.66) for participants with treated and controlled hypertension, as compared with participants without hypertension. There was no significant difference in HbA1c levels between participants with treated but uncontrolled hypertension and those with treated and controlled hypertension.

**Conclusion:**

Data from a large working population, predominantly composed of men, suggest that achievement of HbA1c, BP, and LDL-C targets was less than optimal, especially in younger participants. Uncontrolled dyslipidemia was associated with poor glycemic control. Participants not receiving antihypertensive treatment had higher HbA1c levels.

## Background

Diabetes and its complications are a major public health issue throughout the world [1]. It is estimated that 387 million people had diabetes in 2013, and this number will rise to 592 million by 2035 [2]. In Japan, the prevalence of diabetes has markedly increased in the past few decades [3]. In 2013, there were 7.2 million cases of diabetes in Japan [2], foreboding future growth in premature mortality, morbidity, and economic burden, which are largely associated with its complications. The risk of diabetes complications can be reduced by intensive control of blood glucose [4], blood pressure (BP) [5,6], and blood lipid profile [7]. The American Diabetes Association (ADA) recommends that most adults with diabetes achieve a glycated hemoglobin (HbA1c) < 7.0%, BP < 140/90 mmHg, and low-density lipoprotein cholesterol (LDL-C) < 100 mg/dL [8]. Similarly, the Japan Diabetes Society (JDS) has established targets for the three risk factors for patients with diabetes: HbA1c < 7.0%, BP < 130/80 mmHg, and LDL-C < 120 mg/dL [9].

Despite evidence showing the benefits of simultaneous control of HbA1c, BP, and LDL-C in reducing the risk of diabetes complications and death [10,11], studies from Western [12–14] and Asian [15–17] countries showed that attainment of all three goals simultaneously was low (10–30%). In Japan, there are limited data on treatment and/or achieving rates for patients with diabetes with respect to these risk factors [18,19]. In a clinic- and hospital-based study, 34% of patients had HbA1c < 6.5% and half of the patients had BP < 130/80 mmHg [18]. In a study of health check-up attendants, 44.7% of patients under treatment of anti-diabetic drugs achieved HbA1c (< 7.0%), 51.8% for BP (< 130/80 mmHg), and 58.1% for LDL-C (< 120 mg/dL) [19]. However, these studies did not report the proportion of patients meeting all three targets [18,19]. In addition, no study assessed diabetes control status in the Japanese working population, in which 8.0% of men and 3.3% of women had diabetes [20].

Knowledge about demographic and clinical characteristics associated with glycemic control would be helpful for health-care providers. Younger age, obesity, long duration of diabetes, and co-morbidity are associated with poor glycemic control [21,22]. Use of antihypertensive or lipid-lowering drugs may also influence glycemic control [23–25]. A study in the Netherlands reported lower HbA1c levels in patients with diabetes treated for hypertension compared with patients with diabetes without hypertension [23]. However, it remains elusive, among patients with diabetes treated for hypertension, whether control status of hypertension is additionally associated with glycemic level.

In Japan, employees are required by law to receive an annual health examination including measurement of glycemic status. This provides a valuable opportunity to assess the current control of diabetes in the working population. We conducted a cross-sectional study in participants with diabetes using data of the Japan Epidemiology Collaboration on Occupational Health (J-ECOH) Study. The present study aimed to (1) estimate the prevalence of participants who meet ADA (and JDS) recommendations for HbA1c, BP, and LDL-C and (2) investigate correlates of poor glycemic control.

## Methods

### Survey description

The J-ECOH Study is an ongoing, multicenter, epidemiologic study among employees of 12 companies mainly in the manufacturing industry (electric machinery and apparatus manufacturing; steel, chemical, gas, and non-ferrous metal manufacturing; automobile and instrument manufacturing; plastic product manufacturing; and health care). The investigators of the J-ECOH Study have been collecting several types of health-related data from each participating company, and the present study was based on health check-up data. In Japan, employees are obliged to undergo periodic health examination under the Industrial Safety and Health Act. As of May 2014, 11 of the 12 participating companies provided health check-up data obtained between January 2008 and December 2013 or between April 2008 and March 2014.

Prior to the collection of data, the conduct of the J-ECOH Study was announced in each company by using posters that explained the purpose and procedure of the study. Participants did not provide their verbal or written informed consent to join the study but were allowed to refuse their participation. This procedure conforms to the Japanese Ethical Guidelines for Epidemiological Research, where the procedure of obtaining consent may be simplified for observational studies using existing data [26]. The details of the J-ECOH Study have been described elsewhere [20,27]. The study protocol including consent procedure was approved by the Ethics Committee of the National Center for Global Health and Medicine, Japan (NCGM-G-001140-05).

### Participants

There were a total of 83,234 male and 15,820 female employees in the participating companies in 2013. The majority of employees were male (84%), representing the ratio of male to female employees in the manufacturing industry. Of the employees in the participating companies, about 95% of male and 90% of female employees attended the annual health check-up during the period between January 2013 and December 2013 or between April 2013 and March 2014. In the present study, our analysis was restricted to participants aged 20–69 years who were receiving medical treatment for diabetes, which was defined in two ways: (1) anti-diabetic drug use or non-pharmacological treatment, such as lifestyle modification (five companies, consisting of 76.9% of total study participants, were asked about these) and (2) anti-diabetic drug use (six companies). Of the J-ECOH Study participants, we identified 3,395 diabetic participants under medical treatment. Of these, we excluded those who had missing values for HbA1c, BP, triglyceride (TG), high-density lipoprotein cholesterol (HDL-C), LDL-C, antihypertensive treatment, and lipid-lowering treatment (*n* = 108). Of the remaining 3,287 participants, we excluded participants measured in a non-fasting state (*n* = 217), leaving 3,070 participants (2,854 men and 216 women) for analysis.

### Measurements

The body height, weight, and waist circumference (WC) were measured according to a standard protocol of each company. Body mass index (BMI) was calculated as the weight in kilograms divided by the squared height in meters. WC was measured at the umbilical level using a measuring tape, with the participants in the standing position [28]. Smoking status and medical treatment status for diabetes, hypertension and dyslipidemia were self-reported. Data about medication types and adherence to therapy were not available.

BP was measured with the patient in a sitting position using automatic BP monitors. In most participating companies, BP was measured once, followed by the second measurement if the first measurement was equal to or higher than a certain cutoff defined by the companies (systolic/diastolic BP: 130/85 mmHg, 140/90 mmHg, or 150/90 mmHg). If both first and second measurements were recorded, we used the first one in the present analysis to improve comparability among companies. In two companies in which BP was measured twice for all participants, the lower value was recorded for one company, whereas the first value was recorded in another company. The details of measurement method of BP were shown in S1 Table.

Plasma glucose was measured by the enzymatic or glucose oxidase peroxidative electrode method. HbA1c was measured by using latex agglutination immunoassay, high-performance liquid chromatography, or the enzymatic method. The details of measurement of glucose and HbA1c were shown in S2 Table. In all participating companies, TG, LDL-C, and HDL-C level were measured by the enzymatic method. All laboratories involved in the health check-up in the participating companies have received satisfactory scores (rank A or a score > 95 out of 100) from external quality control agencies.

Hypertension was defined as systolic BP ≥ 140 mmHg, diastolic BP ≥ 90 mmHg, or as receiving medical treatment for hypertension [29]. Dyslipidemia was defined as TG of ≥ 150 mg/dl, LDL-C of ≥ 140 mg/dl, HDL-C of < 40 mg/dl, or as receiving medical treatment for dyslipidemia, based on the criteria for the Japan Atherosclerosis Society [30].

### Treatment goals

For HbA1c, BP, and LDL-C, the goals used for this study were based on the 2015 ADA guidelines (HbA1c < 7.0%, BP < 140/90 mmHg, and LDL-C < 100 mg/dL) [8] and 2013 JDS guidelines (HbA1c < 7.0%, BP < 130/80 mmHg, and LDL-C < 120 mg/dL) [9], respectively. We also examined secondary lipid targets: TG < 150 mg/dL and HDL-C > 40 mg/dL in men and > 50 mg/dL in women [8].

### Statistical analyses

Characteristics of study participants were described in means for continuous variables and percentages for categorical variables by age groups. Trend association was assessed by assigning ordinal numbers to each age group (20–49, 50–59, and 60–69 years old) and was tested using a linear regression analysis and the Cochran–Armitage trend test for continuous and categorical variables, respectively.

We calculated the percentage of participants who met individual and all three (HbA1c, BP, and LDL-C) risk factor goals. For BP and lipids, we identified participants who reported receiving medical treatment at the time of health check-up (lipid-lowering treatment, or antihypertensive treatment). We then examined goal attainment rates for these participants with respect to lipids and BP management.

We analyzed the correlates of poor glycemic control (HbA1c ≥ 8.0%) compared with optimal control defined as HbA1c <7.0% [8,9]. HbA1c of 8.0% is considered as a “take action” threshold in the ADA and JDS guidelines [8,9] and was treated as a cut-off point of poor glycemic control in previous studies [12,19,31,32]. Thus, HbA1c ≥ 8.0% is considered as poor glycemic control in our study. In this analysis, we excluded participants with HbA1c of 7.0–7.9%, which is commonly considered as sub-optimal [12,31,32]. Logistic regression analysis was performed to estimate odds ratio (OR) and 95% confidence interval (CI) of poor glycemic control for age, sex, WC (< 90 cm or ≥ 90 cm for men, < 80 cm or ≥ 80 cm for women), BMI (< 25 kg/m^2^, 25 to < 30 kg/m^2^, and ≥ 30 kg/m^2^), smoking status (current smoker or non-current smoker), dyslipidemia (none, untreated, treated but uncontrolled, treated and controlled), and hypertension (none, untreated, treated but uncontrolled, treated and controlled). We adjusted age, sex, and, worksite in the basic model and additionally adjusted for WC, BMI, smoking status, hypertension, and dyslipidemia in the full model. All statistical analyses were performed using SAS version 9.3 (SAS Institute, Cary, NC, USA), and two-sided *P* < 0.05 was considered statistically significant.

## Results

### Participant characteristics

Of 3,070 participants with diabetes, 6.2% were female. The mean age was 53.7 ± 7.3 years. The characteristics of participants by age group are shown in Table 1. The prevalence of smoking was higher in the younger age group (*P* for trend < 0.001). The mean WC, BMI, HbA1c, fasting plasma glucose, diastolic BP, LDL-C, and TG were higher in younger participants, whereas systolic BP and HDL-C were higher in older participants (*P* for trend < 0.001). The prevalence of hypertension and the proportion of participants under hypertension treatment among those with hypertension increased with advancing age (*P* for trend < 0.001). The prevalence of dyslipidemia was higher in the younger age group (*P* for trend < 0.001). No age difference was found in the prevalence of lipid-lowering treatment.

**Table 1:** Characteristics of participants with diabetes.

	Age (years)			Total
	20–49	50–59	60–69	
N	869	1470	731	3,070
Female, %	7.3	5.7	5.9	6.2
Current smoker^¶^, %	43.9	39.9	32.9*	39.4
WC (cm)	94.6±13.0	89.7±9.9	87.1±8.9*	90.5±11.0
BMI (kg/m^2^)	28.3±5.4	25.9±4.1	24.7±3.4*	26.3±4.5
HbA1c (%)	7.7±1.5	7.3±1.2	7.1±1.0*	7.3±1.2
FPG (mg/dL)	152.4±44.5	146.5±36.8	141.5±32.4*	147.0±38.4
**BP**				
SBP (mmHg)	127.4±14.1	127.7±14.9	129.8±15.4*	128.1±14.8
DBP (mmHg)	81.2±10.0	80.4±9.7	78.5±8.6*	80.2±9.6
Hypertension, %	48.2	57.1	63.3*	56.0
Anti-hypertension treatment^†^, %	79.7	88.7	88.3*	86.4
**Lipids**				
LDL-C (mg/dL)	122.7±30.1	116.3±30.3	113.2±26.4*	117.3±29.6
TG (mg/dL)	169.3±143.1	146.5±120.2	130.1±78.2*	149.0±119.8
HDL-C (mg/dL)	48.8±11.8	52.4±13.9	54.1±14.2*	51.8±13.5
Dyslipidemia	74.2	69.2	60.3*	68.5
Lipid-lowering treatment^‡^, %	53.3	62.4	56.9	58.5

Data was expressed as mean ±SD or as percentages.

*P for trend < 0.001.

¶ Data were available for 3006 participants.

†The denominator is the total number of people with hypertension.

‡The denominator is the total number of people with dyslipidemia.

WC: waist circumference, BMI: body mass index, FPG: fasting plasma glucose, SBP: systolic blood pressure, DBP: diastolic blood pressure, LDL-C: low-density lipoprotein cholesterol, TG: triglyceride, HDL-C: high-density lipoprotein cholesterol.

### Prevalence of meeting risk factor targets

Table 2 shows the prevalence of meeting risk factor targets in participants with diabetes. Of the participants, 44.9% met the target for HbA1c (< 7.0%). Approximately three-fourths and one-quarter of participants met ADA targets for BP (< 140/90 mmHg) and LDL-C (< 100 mg/dL), respectively. Approximately one-third and one-half of participants met JDS targets for BP (< 130/80 mmHg) and LDL-C (< 120 mg/dL), respectively. The proportion of attainment of all three (HbA1c, BP, and LDL-C) target achievements was 11.2% by the ADA recommendations and 10.8% by the JDS recommendations. Approximately two-thirds had TG values < 150 mg/dL, and four-fifths had HDL-C > 40 mg/dL (50 mg/dL for women). The prevalence of HbA1c, LDL-C, TG, and HDL-C control increased with age (*P* for trend < 0.001).

**Table 2:** Prevalence of meeting risk factor targets in participants with diabetes.

	Age (years)			Total
	20–49	50–59	60–69	
HbA1c < 7.0%, %	36.6	46.1	52.3*	44.9
**BP, %**				
ADA (< 140/90 mmHg) ^¶^	75.4	77.4	76.6	76.6
JDS (< 130/80 mmHg) ^§^	35.8	36.4	36.8	36.3
**LDL-C, %**				
ADA (< 100 mg/dL) ^¶^	21.5	28.1	31.9*	27.1
JDS (< 120 mg/dL) ^§^	48.2	58.1	61.7*	56.2
**HbA1c, BP and LDL-C**^**†**^**, %**				
ADA (HbA1c, BP and LDL-C) ^¶^	7.0	12.8	13.1*	11.2
JDS (HbA1c, BP and LDL-C) ^§^	7.8	11.2	13.4*	10.8
TG < 150 mg/dL^¶^, %	58.3	66.5	72.0*	65.5
HDL-C > 40 mg/dL^¶^ ^‡^, %	73.1	81.8	83.5*	79.7

*P for trend < 0.001.

¶ American Diabetes Association Standards of medical care in diabetes– 2015.

§ Japan Diabetes Society Treatment Guide for Diabetes– 2013.

†Meeting targets for all three risk factors simultaneously.

‡50 mg/dL for women.

BP: blood pressure, LDL-C: low-density lipoprotein cholesterol, TG: triglyceride, HDL-C: high-density lipoprotein cholesterol.

Table 3 shows goal attainment rates for participants who were receiving antihypertensive or lipid lowering treatment. Of participants with antihypertensive treatment (*n* = 1,488), 67.5% and 24.3% met ADA target for BP (< 140/90 mmHg) and JDS target for BP (< 130/80 mmHg), respectively. Of participants with lipid-lowering treatment (*n* = 1,230), 29.4% and 57.0% met ADA target for LDL-C (< 100 mg/dL) and JDS target for LDL-C (< 120 mg/dL), respectively. Approximately three-fifths had TG values <150 mg/dL, and three-fourths had HDL-C > 40 mg/dL (50 mg/dL for women). The prevalence rates of BP, LDL-C, TG, and HDL-C control increased with age (*P* for trend < 0.05).

**Table 3:** Prevalence of meeting risk factor targets in participants receiving blood pressure- and lipid- lowering treatment.

	Age (years)			Total
	20–49	50–59	60–69	
**Anti-hypertension treatment, n**	334	745	409	1,488
ADA (BP < 140/90 mmHg) ^¶^, %	61.4	68.2	71.4*	67.5
JDS (BP < 130/80 mmHg)^§^, %	18.9	24.4	28.6*	24.3
**Lipid-lowering treatment, n**	344	635	251	1,230
ADA (LDL-C < 100 mg/dL)^¶^, %	27.0	28.4	35.5*	29.4
JDS (LDL-C < 120 mg/dL)^§^, %	50.0	57.3	65.7*	57.0
TG < 150 mg/dL^¶^, %	50.9	59.8	68.5*	59.1
HDL-C > 40 mg/dL^¶^ ^†^, %	70.0	80.8	80.5*	77.6

*P for trend < 0.05.

¶ American Diabetes Association Standards of medical care in diabetes– 2015.

§ Japan Diabetes Society Treatment Guide for Diabetes– 2013.

†50 mg/dL for women.

BP: blood pressure, LDL-C: low-density lipoprotein cholesterol, TG: triglyceride, HDL-C: high-density lipoprotein cholesterol.

### Correlates of poor glycemic control

In our study, 721 participants had HbA1c level ≥ 8.0%. Associations of demographic and clinical characteristics with poor glycemic control are presented in Table 4. Younger age is significantly associated with poor glycemic control, with the OR being 2.02 (95% CI, 1.52–2.70) and 1.33 (1.02–1.72) for the age groups of 20–49 years and 50–59 years, respectively, as compared with 60–69 years old. Larger WC and BMI are associated with poor glycemic control. The OR of poor glycemic control for current smoking versus non-current smoking was 1.28 (1.05–1.57).

**Table 4:** Factors related with poor glycemic control (HbA1c ≥ 8.0%).

		OR (95% CI)	
	N	Basic model^¶^	Full model^Ҹ^
**Age (years)**			
20–49	614	3.15 (2.42–4.11)	2.02 (1.52–2.70)
50–59	991	1.60 (1.24–2.05)	1.33 (1.02–1.72)
60–69	494	Referent	Referent
**Sex**			
Male	1,958	Referent	Referent
Female	141	0.94 (0.65–1.35)	0.95 (0.64–1.40)
**WC**^†^ **(cm)**			
< 90	1,050	Referent	Referent
≥ 90	1,049	1.48 (1.22–1.79)	1.24 (0.94–1.64)
**BMI (kg/m**^**2**^**)**			
< 25	905	Referent	Referent
25– < 30	844	1.41 (1.15–1.74)	1.25 (0.95–1.64)
≥ 30	350	1.69 (1.29–2.22)	1.60 (1.09–2.33)
**Current smoker**			
No	1,268	Referent	Referent
Yes	787	1.32 (1.09–1.60)	1.28 (1.05–1.57)
**Hypertension**			
None	921	Referent	Referent
Untreated	168	1.34 (0.95–1.88)	1.22 (0.86–1.74)
Treated but uncontrolled	748	0.68 (0.55–0.84)	0.58 (0.46–0.73)
Treated and controlled^§^	262	0.52 (0.37–0.72)	0.47 (0.33–0.66)
**Dyslipidemia**			
None	662	Referent	Referent
Untreated	604	1.90 (1.49–2.42)	1.70 (1.32–2.19)
Treated but uncontrolled	544	1.79 (1.39–2.30)	1.70 (1.31–2.20)
Treated and controlled^£^	289	0.87 (0.62–1.21)	0.89 (0.64–1.25)

¶ Age was adjusted by sex and worksite; sex was adjusted by age and worksite; WC, BMI, current smoker, dyslipidemia, and hypertension were adjusted by age, sex, and worksite.

Ҹ All variables including age, sex, worksite, WC, BMI, current smoker, dyslipidemia, and hypertension were entered.

†80 cm for women.

§ Controlled hypertension was defined as systolic blood pressure < 130 mmHg and diastolic blood pressure < 80 mmHg, based on the 2014 Japanese Society of Hypertension Guidelines for the Management of Hypertension.

£ Controlled dyslipidemia was defined by TG of < 150 mg/dL, LDL-C of < 120 mg/dL, and HDL-C of ≥ 40 mg/dL, based on the criteria for the Japan Atherosclerosis Society.

Participants with untreated hypertension had a non-significant 22% higher odds (OR, 1.22; 95% CI, 0.86–1.74) for poor glycemic control compared with participants without hypertension. In contrast, participants with treated hypertension, irrespective of BP control, were less likely to have poor glycemic control than participants without hypertension. The OR of having poor glycemic control was 0.58 (0.46–0.73) for participants with treated but uncontrolled hypertension and 0.47 (0.33–0.66) for participants with treated and controlled hypertension. No significant difference was observed in HbA1c levels between the two groups.

Uncontrolled dyslipidemia was associated with poor glycemic control, with the OR being 1.70 (1.32–2.19) and 1.70 (1.31–2.20) for participants with untreated dyslipidemia and participants with treated but uncontrolled dyslipidemia, respectively, as compared with participants without dyslipidemia. There was no such association for participants with treated and controlled dyslipidemia (OR, 0.89; 95% CI 0.64–1.25).

## Discussion

In the present study among a large working population in Japan, the percentages of participants who met ADA (and JDS) targets were 44.9% (44.9%) for HbA1c, 76.6% (36.3%) for BP, 27.1% (56.2%) for LDL-C, and 11.2% (10.8%) for simultaneous control of all three risk factors. Younger age, obesity, smoking, and uncontrolled dyslipidemia were associated with increased odds of poor HbA1c control, whereas antihypertensive treatment was associated with reduced odds.

### HbA1c, BP, and LDL-C control

Regarding HbA1c, less than half of the participants in our study reached the HbA1c target. This finding is similar to that in a previous Japanese study of patients who received anti-diabetic drugs (HbA1c < 7.0%, 44.7%) [19]. Similar achievement rates for HbA1c have also been reported from other countries [12,15,33]. The U.S. National Health and Nutrition Examination Survey (NHANES) 2007–2010 reported that 52.5% of adults diagnosed with diabetes achieved HbA1c < 7.0% [12]. In the Korean National Health and Nutrition Examination Survey, 49.1% of adults diagnosed with diabetes had HbA1c < 7.0% in 2010 [15]. A national survey in China showed that 39.7% of patients treated for diabetes had optimal glycemic control in 2010 [33]. Although these studies differed in their sample sizes, population, and survey period, the results showed that glycemic control is a challenge in both Asian and Western countries.

As for BP, only one-third of participants achieved the JDS goal of BP (< 130/80 mmHg). Similar low achievement rates for BP (< 130/80 mmHg) goal have also been reported from other Asian countries [15,17]. A joint research among seven Asian countries showed that 32.3% of patients with diabetes enrolled through physicians met target for BP < 130/80 mmHg in 2007–2009 [17]. The Japanese Society of Hypertension emphasizes that the target BP level for patients with hypertension and diabetes should be less than 130/80 mmHg because strict BP control is necessary in patients with hypertension and diabetes for preventing cardiovascular disease, especially stroke [29]. Our further analysis showed that only approximately 20% of participants with hypertension and diabetes achieved BP goal (< 130/80 mmHg). This indicates that a large proportion of participants with hypertension and diabetes are at high risk of developing cardiovascular disease, including stroke.

For LDL-C, approximately one-quarter of participants achieved the ADA LDL-C (< 100 mg/dL) goal and one-half achieved the JDS goal of LDL-C (< 120 mg/dL). The U.S. NHANES 2007–2010 survey showed that more than half of the patients with diabetes achieved the ADA LDL-C goal [12]. In Korea, nearly half of the patients with diabetes reached the ADA LDL-C goal [15]. The relatively low achievement rates for LDL-C goal (< 100 mg/dL) in Japanese patients may be due to the less stringent JDS LDL-C goal (< 120 mg/dL) [10] and/or suboptimal management of dyslipidemia in patients with diabetes. In our study, only half of participants with dyslipidemia were receiving lipid-lowering treatment, and 29.4% of those treated for dyslipidemia achieved the ADA LDL-C goal.

In the present study, only one in ten met all three targets. This finding is comparable to those in Asian studies [15–17], in which approximately 10% of patients met all three targets. To reduce the risk of future complications, there is a need to improve the comprehensive management of diabetes in the Japanese working population.

### Correlates of poor glycemic control

We analyzed characteristics that could be associated with the poor control of HbA1c (≥ 8.0%). Younger participants were less likely to meet risk factor goals and had a poorer glycemic control, as reported in previous studies [12,22]. It is speculated that younger patients may be busy with their job and have less time to comply with a healthy lifestyle and treatment [22]. In addition, younger patients may not perceive the need for good diabetic control because their quality of life has not yet been affected by diabetic complications, which take a number of years to develop [34]. Consistent with previous studies [22,35], obesity and smoking were related with poor glycemic control. Increased insulin resistance occurs in smokers with and without diabetes [36,37].

Participants with untreated hypertension had a nonsignificant 22% higher odds of poor glycemic control compared with participants without hypertension. In contrast, participants receiving antihypertensive treatment (regardless of whether BP was controlled or not) were more likely to have optimal HbA1c control compared with participants without hypertension. The reason for this is unclear. One possible explanation might be that some antihypertensive drugs have beneficial effects on glucose metabolism [38]. The Japanese Society of Hypertension recommends the use of angiotensin-converting enzyme inhibitors (ACE inhibitors) and angiotensin receptor blockers (ARBs), which enhance insulin sensitivity [39], for patients with diabetes and hypertension [29]. A study in Japan showed that 33% of patients with hypertension and diabetes were taking ACE inhibitors and/or ARBs [40].

Our results indicated that participants with uncontrolled dyslipidemia (untreated or treated but uncontrolled) were more likely to have poor HbA1c control. In line with our findings, previous studies also showed that poor lipid profiles were associated with poor glycemic control [41,42]. The mechanisms have not been completely clarified. The higher HbA1c levels in patients with abnormal lipids may partly be due to the adverse effect of free fatty acids on insulin sensitivity [43]. Further study is needed to clarify the role of dyslipidemia and its treatment in diabetes control.

### Limitations

Our real-life data reflect actual treatment status in participants with diabetes in the working population. However, several limitations need to be considered. First, because the majority of study participants were employees of large companies, caution should be exercised in generalizing the present findings to workers in smaller-sized companies or non-working populations. Second, because the majority of the participants were male employees of manufacturing companies, the results thus may not be generalizable to female and employees in other industries. Third, the methods of blood glucose and HbA1c measurements differed among the companies. Given satisfactory results of external quality control in all of the participating companies, however, measurement bias is unlikely. We used the first BP reading in analysis to improve comparability across companies. This might have led to some overestimation of poor BP control. Fourth, we did not have detailed data about medication types and patients’ adherence to medications for diabetes, hypertension, and dyslipidemia. This has limited our interpretation of the results. Fifth, the control rates of hypertension and dyslipidemia may be somewhat underestimated because some patients might have skipped their medications on the day of health check-up. Finally, a causal relationship between dyslipidemia, hypertension, and glycemic control cannot be established in this cross-sectional study.

Data from a Japanese working population, predominantly composed of men, suggest that achievement of management targets for HbA1c, BP, and LDL-C is less than optimal, especially in younger participants. Uncontrolled dyslipidemia was associated with poor glycemic control. Participants not receiving antihypertensive treatment have higher HbA1c levels. The control of blood glucose, BP, and lipid should be strengthened to reduce the cardiovascular risk of patients with diabetes in Japan.

## Supporting Information

S1 Table Measurement of blood pressure according to participating companies. (DOCX)

S2 Table Measurement of glucose and HbA1c according to participating companies. (DOCX)
