# Supplementary material for: Hba1c, Blood Pressure, and Lipid Control in People with Diabetes: Japan Epidemiology Collaboration on Occupational Health Study
Source: PLoS One. 2016 Jul 20;11(7):e0159071. doi: 10.1371/journal.pone.0159071 (PMC4954688; doi:10.1371/journal.pone.0159071)
Supplement: S1 Table — (DOCX) [file pone.0159071.s001.docx]

S1 Table Measurement of blood pressure according to participating companies

| Company | First measurement | Second measurement | Notes | Analysis data |
| --- | --- | --- | --- | --- |
| A | Yes | Conditional | If first BP ≥140/90 mmHg, then take a second measurement | If both first and second measurements were recorded, we used the first one for the present analysis. |
| B | Yes | Conditional | If first BP ≥130/85 mmHg, then take a second measurement | If both first and second measurements were recorded, we used the first one for the present analysis. |
| C | Yes | Conditional | If first BP ≥140/90 mmHg, then take a second measurement | If both first and second measurements were recorded, we used the first one for the present analysis. |
| D | Yes | Conditional | If first BP ≥140/90 mmHg, then take a second measurement | If both first and second measurements were recorded, we used the first one for the present analysis. |
| E | Yes | Yes | Only lower values were recorded for each people | Lower values were provided by the company. |
| F | Yes | Yes | Both measurements were recorded | First measurement was used in the present analysis. |
| G | Yes | Conditional | If first BP ≥130/85 mmHg, then take a second measurement | If both first and second measurements were recorded, we used the first one for the present analysis. |
| H | Yes | Conditional | If first BP ≥130/85 mmHg, then take a second measurement | If both first and second measurements were recorded, we used the first one for the present analysis. |
| I | Yes | Conditional | If first BP ≥130/85 mmHg, then take a second measurement | If both first and second measurements were recorded, we used the first one for the present analysis. |
| J | Yes | Conditional | If first BP ≥150/90 mmHg, then take a second measurement | If both first and second measurements were recorded, we used the first one for the present analysis. |
| K | Yes | Conditional | If first BP ≥140/90 mmHg, then take a second measurement | If both first and second measurements were recorded, we used the first one for the present analysis. |
