# Supplementary material for: Hba1c, Blood Pressure, and Lipid Control in People with Diabetes: Japan Epidemiology Collaboration on Occupational Health Study
Source: PLoS One. 2016 Jul 20;11(7):e0159071. doi: 10.1371/journal.pone.0159071 (PMC4954688; doi:10.1371/journal.pone.0159071)
Supplement: S2 Table — (DOCX) [file pone.0159071.s002.docx]

S2 Table Measurement of glucose and HbA1c according to participating companies

| Inspection item | Company | Methods | Reagent (Manufacturer) | Measuring equipment (Manufacturer) |
| --- | --- | --- | --- | --- |
| Glucose | A | Glucose oxidase peroxidative electrode method | Reagent for GA-1170 (Arkray Inc.) | ADAMS glucose GA-1170 (Arkray Inc.) |
|  | B | Enzymatic method (Hexokinase-UV method) | Pureauto S GLU (Sekisui Medical Co., Ltd.) | BM9030 (JEOL) |
|  | C | Enzymatic method (Hexokinase-UV method) | Quick-auto-Ⅱ-GLU-HK (Shino-Test Corp.) | BM9030 (JEOL) |
|  | D | Enzymatic method (Hexokinase-UV method) | GLU-S (Denka Seiken Co., Ltd.) | BM1250 (JEOL) |
|  | E | Enzymatic method (Glucose oxidase method) | Cica liquid GLU J (Kanto Chemical Co., Inc.) | BM9030 (JEOL) |
|  | F | Enzymatic method (Hexokinase-UV method) | Quick-auto-Ⅱ-GLU-HK (Shino-Test Corp.) | BM9030 (JEOL) |
|  | G | Enzymatic method (Glucose oxidase method) | Cica liquid GLU J (Kanto Chemical Co., Inc.) | AU5400 (BECKMAN COULTER) |
|  | H | Enzymatic method (Glucose oxidase method) | IATRO-LQ GLU (Mitsubishi Chemical Medience Corp.) | JCA-BM1650 (JEOL) |
|  | I | Enzymatic method (Hexokinase-UV method) | Pureauto S GLU-R (Sekisui Medical Co., Ltd.) | BM9130 (JEOL) |
|  | J | Enzymatic method (Hexokinase-UV method) | L-type WAKO GLU2 (Wako Chemical Co., Inc.) | AU640 |
|  | K | Enzymatic method (Hexokinase-UV method) | L-type WAKO GLU2 (Wako Chemical Co., Inc.) | AU640 |
|  |  |  |  |  |
| HbA1c | A | HPLC method | Reagent for G9 (Arkray Inc.) | ADAMS-HA8160 (Arkray Inc.) |
|  | B | Latex agglutination immunoassay | RAPIDIA Auto HbA1c-L (Fujirebio, Inc.) | BM9030 (JEOL) |
|  | C | Latex agglutination immunoassay | Determiner HbA1c (Kyowa Medex Co., Ltd.) | BM9030 (JEOL) |
|  | D | HPLC method | Reagent for HLC-723G8 (Tosoh) | HLC-723G8 (Tosoh) |
|  | E | Latex agglutination immunoassay | Determiner HbA1c (Kyowa Medex Co., Ltd.) | BM9030 (JEOL) |
|  | F | Latex agglutination immunoassay | Determiner HbA1c (Kyowa Medex Co., Ltd.) | BM9030/BM9031 (JEOL) |
|  | G | Latex agglutination immunoassay | RAPIDIA Auto HbA1c-L (TFB, Inc.) | JCA-BM9130 (JEOL) |
|  | H | Enzymatic method | CinQ HbA1c (Mitsubishi Chemical Medience Corp.) | JCA-BM9030 (JEOL) |
|  | I | Enzymatic method | Nordia-N HbA1c (Sekisui Medical Co., Ltd.) | BM9130 (JEOL) |
|  | J | Latex agglutination immunoassay | RAPIDIA Auto HbA1c-L (Fujirebio, Inc.) | AU640 |
|  | K | Latex agglutination immunoassay | RAPIDIA Auto HbA1c-L (Fujirebio, Inc.) | AU640 |
